# A Nested Case–Control Study on the Risk of Surgical Site Infection After Thyroid Surgery

**DOI:** 10.1007/s00268-018-4492-2

**Published:** 2018-02-22

**Authors:** F. A. Salem, M. Almquist, E. Nordenström, J. Dahlberg, O. Hessman, C. I. Lundgren, A. Bergenfelz

**Affiliations:** 1 Department of Clinical Sciences, Lund University, Lund, Sweden; 2 Department of Endocrine Surgery, Sahlgrenska University Hospital, Gothenburg, Sweden; 3 Department of Endocrine Surgery, Uppsala University Hospital, Uppsala, Sweden; 4 Department of Endocrine Surgery, Karolinska University Hospital, Stockholm, Sweden; 5 Skåne University Hospital, 221 85 Lund, Sweden

## Abstract

**Introduction:**

It is unclear if antibiotic prophylaxis reduces the risk of surgical site infection (SSI) in thyroid surgery. This study assessed risk factors for SSI and antibiotic prophylaxis in subgroups of patients.

**Method and design:**

A nested case–control study on patients registered in the Swedish National Register for Endocrine Surgery was performed. Patients with SSI were matched 1:1 by age and gender to controls. Additional information on patients with SSI and controls was queried from attending surgeons using a questionnaire. Risk factors for SSI were evaluated by logistic regression analysis and presented as odds ratio (OR) with 95% confidence interval (CI).

**Results:**

There were 9494 operations; 109 (1.2%) patients had SSI. Patients with SSI were older (median 53 vs. 49 years) than patients without SSI *p* = 0.01 and more often had a cancer diagnosis 23 (21.1%) versus 1137 (12.1%) *p* = 0.01. In the analysis of patients with SSI versus controls, patients with SSI more often had post-operative drainage 68 (62.4%) versus 46 (42.2%) *p* = 0.01 and lymph node surgery 40 (36.7%) versus 14 (13.0%) *p* < 0.01, and both were independent risk factors for SSI, drain OR 1.82 (CI 1.04–3.18) and lymph node dissection, OR 3.22 (95% CI 1.32–7.82). A higher number of 26(62%) patients with independent risk factors for SSI and diagnosed with SSI did not receive antibiotic prophylaxis. Data were missing for 8 (31%) patients.

**Conclusion:**

Lymph node dissection and drain are independent risk factors for SSI after thyroidectomy. Antibiotic prophylaxis might be considered in patients with these risk factors.

## Introduction

Although surgical site infection (SSI) after thyroid surgery is uncommon, it may lead to further complications and increased healthcare cost [1–3].

The frequency of SSI after thyroid surgery has been estimated to be 0.3–2.9% [1, 4–8].

Surgical site infection may manifest as wound cellulitis and treated with oral antibiotics or as infected seroma which may require drainage and culture-directed treatment with intravenous antibiotics [9].

In studies published during the last two decades, prolonged operation time, use of drains, reoperation due to bleeding and concomitant lymph node dissection have been reported as risk factors for SSI in thyroid surgery [1, 4, 10, 11].

The use of preoperative antibiotic prophylaxis is generally not recommended due to the low incidence of SSI after thyroid surgery [1, 12–14]. However, in practice this routine varies [15, 16]. Whether or not there is a subgroup of patients with higher risk of SSI where prophylactic antibiotics could be considered is at present unclear. Results from a multicentre retrospective observational study on 2926 patients who had underwent thyroid surgery showed that antibiotic prophylaxis did not decrease the incidence of SSI [17].

In a single-centre randomized clinical trial (RCT), Uruno et al. [13] showed that antibiotic prophylaxis did not protect the patient from SSI in thyroid surgery. However, all patients with reported SSI had post-operative drains and underwent lymph node dissection, with one exception.

This retrospective multicentre study, therefore, aimed to investigate risk factors for SSI and the effect of antibiotic prophylaxis in a national cohort of operated patients undergoing thyroid surgery. Data were registered in the national quality register for endocrine surgery, and additional information was queried from attending surgeons by questionnaires. The course of treatment of patients with SSI is also described.

## Methods

### Scandinavian quality register for thyroid, parathyroid and adrenal surgery

Data were collected from the Scandinavian Quality Register for Thyroid, Parathyroid and Adrenal Surgery (SQRTPA), which is the recognized quality register in this medical field in Sweden. The registry is endorsed by the Swedish Association of Endocrine Surgeons and Swedish Association of Otorhinolaryngology and Head and Neck Surgery. Data are registered prospectively by the attending surgeons.

Operations performed for thyroid disease registered in the database 2004–2010 were included in the study. Data were extracted from the registry in March 2012.

Surgical site infection (SSI) was defined as a local wound complication registered in SQRPTA by the attending surgeon.

### Matching cohort

Since some additional information, among those ongoing medication, the use of antibiotic prophylaxis, co-morbidities, body mass index, smoking habits, the use of drain and the course of treatment for SSI were not registered in SQRTPA, patients with SSI were matched 1:1 by age and gender to controls. This additional information was queried by questionnaire, which was sent to the attending surgeons in 27 departments. Data from the questionnaires were collected and merged with the data from SQRTPA for further analysis. Through the same questionnaire, it was confirmed that data entry in the SQRTPA was correct.

### Analysis

Two different types of analyses were performed.

1. Patients with SSI were compared with patients without SSI in the SQRTPA cohort in order to assess the impact of age and gender as risk factors.
2. Patients with SSI were analysed to controls matched 1:1 as a nested case–control study to evaluate additional possible risk factors for SSI which were not available in the SQRTPA.

Patients with independent risk factors for SSI in the multivariable analysis in the case–control cohort were analysed regarding the use of antibiotic prophylaxis.

### Statistics

Risk factors for patients with SSI were evaluated using univariable and multivariable logistic regression and presented as odds ratio (OR) and 95% confidence interval (CI). Continuous variables are reported as median and interquartile range (IQR) and analysed using Wilcoxon rank-sum test. Categorical variables were analysed using Chi-square test.

A *p* value < 0.05 was considered significant.

Variables with *p* < 0.10 and with missing values less than 50% (age, gender, indication for operation, lymph node dissection, post-operative bleeding, operation time, specimen weight, drainage and diabetes) were included in the multivariable logistic regression model.

For the statistical analysis, STATA/IC version 12.0 was used.

### Ethical issues

The study was approved by the regional ethical committee for clinical trials (DNR 2011/740).

## Results

During the study period, 9494 operations were registered. A total of 236 (2.5%) patients were operated with compartment-oriented lymph node dissection only. Surgical site infection was reported in 109 (1.2%) patients. All 109 patients with SSI were successfully matched with control patients from the cohort. The response rate for the questionnaire was 96% (210 answers out of 218 sent questionnaires).

### Patients with and without surgical site infection in the total cohort

Patients with SSI were older with a median (IQR) age of 53 (41–65) versus 49 (37–62) years compared with patients without SSI, *p* = 0.01, and more often had a histological diagnosis of malignancy 23 (21.1%) versus 1137 (12.1%), respectively, *p* = 0.01. Patients with SSI were more often subjected to lymph node dissection 40 (36.7%) versus 1453 (15.3%), *p* < 0.01, and had a higher incidence of post-operative bleeding 7 (6.5%) versus 167 (1.8%), *p* < 0.01. Patients with SSI also had a higher median specimen weight of 65 g (24–125) versus 37 g (20–82) *p* = 0.02 and a somewhat increased risk for an operation time of more than 120 min 40 (36.7%) versus 2945 (31.4%) in control patients, although missing values for operation time have to be accounted for *p* < 0.01 (Table 1).

**Table 1:** Clinical characteristics of patients registered in the Swedish National Registry for Endocrine Surgery, with and without post-operative surgical site infection

Characteristics	No SSI *n* = 9385 (%)	SSI *n* = 109 (%)	*P* value
Age			
*Median/IQR*	*49/37*–*62*	*53/41*–*65*	0.01
Gender			
Male	1804 (19.2)	29 (26.6)	0.05
Female	7581 (80.8)	80 (73.4)	
Indication for operation			
Compression symptom	3627 (38.6)	47 (43.1)	0.01
Malignancy	1137 (12.1)	23 (21.1)	
Excluding malignancy	2290 (24.4)	20 (18.3)	
Thyrotoxicosis	2220 (23.7)	17 (15.6)	
Other(*)	111 (1.2)	2 (1.8)	
Type of operation			
Total thyroidectomy	3944 (42.0)	43 (39.5)	0.70
Hemithyroidectomy	4500 (48.0)	54 (49.5)	
Other(**)	711 (7.6)	6 (5.5)	
Missing value	230 (2.4)	6 (5.5)	
Lymph node dissection			
Yes	1453 (15.3)	40 (36.7)	<0.01
No	7932 (83.5)	69 (63.3)	
Reoperation due to post-operative bleeding			
Yes	167 (1.8)	7 (6.5)	<0.01
No	9218 (98.2)	102 (93.5)	
Operation time (min)			
<60	492(5.2)	1(0.9)	<0.01
61–120	3085(32.9)	20(18.4)	
>120	2945(31.4)	40(36.7)	
Missing value	2863 (30.5)	48 (44.0)	
Previous thyroid surgery			
Yes	1053 (11.2)	13 (11.9)	0.82
No	8332 (88.8)	96 (88.0)	
Specimen weight (g)			
*Median/IQR*	*37/20*–*82*	*65/24*–*125*	0.02
Missing value	2522 (26.9)	42 (38.5)	
Substernal gland			
Yes	798 (8.5)	14 (12.8)	0.10
No	8587 (91.5)	95 (87.2)	

Data are reported in percentage for categorical variables and median (interquartile range) for quantitative ones

Median/IQR is presented in italics

*SSI* surgical site infection, *IQR* interquartile range

(*) Unknown indication registered

(**) Include unilateral and bilateral resections of the thyroid gland

The multivariable logistic regression showed that lymph node dissection, OR 4.10 (95% CI 1.88–8.91), *p* < 0.01, was an independent risk factor for SSI (Table 2).

**Table 2:** Multivariable analysis of risk factors for surgical site infection in patients registered in the Swedish National Registry for Endocrine Surgery

Variables	Odds ratio	Confidence interval	*P* value
Age (year)	1.01	1.00–1.03	0.05
Gender			
Female	1.00		
Male	1.12	0.63–2.01	0.68
Indication for operation			
Compression symptoms	1.00		
Malignancy	0.47	0.18–1.22	0.12
Excluding malignancy	0.39	0.17–0.88	0.02
Thyrotoxicosis	0.94	0.49–1.79	0.85
Lymph node dissection			
No	1.00		
Yes	4.10	1.88–8.91	<0.01
Post-operative bleeding			
No	1.00		
Yes	2.24	0.68–7.32	0.18
Operation time (min)			
<60	1.00		
61–120	1.88	0.23–13.87	0.56
>120	3.34	0.44–25.16	0.24
Missing	3.19	0.04–24.21	0.26
Specimen weight (g)	1.00	0.99–1.00	0.61

Nested case–control analysis of patients with surgical site infection compared to controls.

After matching for gender and age, patients with SSI were more often operated due to malignancy 23 (21.1%) versus 8 (7.3%) patients among controls, *p* = 0.03, and more often underwent concomitant lymph node dissection 40 (36.7%) versus 14 (13.0%) patients, *p* < 0.01. Patients with SSI were also more often treated with post-operative drains 68 (62.4%) versus 46 (42.2%) patients, *p* = 0.01 (Table 3).

**Table 3:** Clinical characteristics of the patients with surgical site infection and control patients using data from Swedish National Registry for Endocrine Surgery and questionnaire data from participating departments

Characteristics	SSI *n* = 109 (%)	Controls *n* = 109 (%)	*P* value
Age			
*Median/IQR*	*53/41*–*65*	*53/41*–*65*	1.00
Gender			
Male	29 (26.6)	29 (26.6)	1.00
Female	80 (73.4)	80 (73.4)	
Indication for surgery			
Compression symptoms	47 (43.1)	53 (48.6)	0.03
Malignancy	23 (21.1)	8 (7.3)	
Excluding malignancy	20 (18.3)	19 (17.4)	
Thyrotoxicosis	17 (15.6)	28 (25.7)	
Other	2 (1.8)	1 (0.9)	
Type of operation			
Total thyroidectomy	43 (39.5)	46 (42.2)	0.30
Hemithyroidectomy	54 (49.5)	56 (51.3)	
Other	6 (5.5)	4 (3.7)	
Missing value	6 (5.5)	3 (2.8)	
Lymph node dissection			
Yes	40 (36.7)	14 (13.0)	<0.01
No	69 (63.3)	95 (87.0)	
Previous thyroid surgery			
Yes	13 (12.0)	13 (12.0)	1.00
No	96 (88.0)	96 (88.0)	
Operation time (min)			0.03
<60	1(0.9)	5(4.6)	
61–120	20(18.4)	34(31.2)	
>120	40(36.7)	36(33.0)	
Missing value	48 (44.0)	34 (31.2)	
Specimen weight (g)	*65/24*–*125*	*54.5/26*–*123*	
Missing value	42 (38.5)	26 (29.0)	0.93
Substernal goitre			
Yes	14 (12.8)	15 (13.6)	0.84
No	95 (87.2)	94 (86.4)	
Reoperation due to post-operative bleeding			
Yes	7 (6.4)	2 (1.8)	0.09
No	102 (93.6)	107 (98.2)	
Cortisone medication*			
Yes	4 (3.6)	6 (5.4)	0.12
No	99 (91.0)	89 (81.6)	
Missing value	6 (5.4)	14 (13.0)	
Prophylactic antibiotics			
Yes	3 (2.8)	6 (5.5)	0.50
No	75 (69.0)	94 (86.2)	
Missing value	31 (28.2)	9 (8.3)	
Diabetes			
Yes	9 (8.3)	2 (1.8)	0.06
No	95 (87.0)	98 (89.9)	
Missing value	5 (4.7)	9 (8.2)	
Drainage			
Yes	68 (62.4)	46 (42.2)	0.01
No	34 (31.2)	56 (51.4)	
Missing	7 (6.4)	7 (6.4)	
BMI	*27/23*–*29*	*26/23*–*29*	
Missing value	79 (72.4)	46 (42.2)	0.88
Smoker or ex-smoker			
Yes	10(9.1)	14(12.8)	0.02
No	18(16.5)	33(30.2)	
Missing value	81(74.4)	62(57.0)	

Data are reported in percentage for categorical variables and median (interquartile range) for quantitative ones

Median/IQR is presented in italics

*SSI* surgical site infection, *BMI* body mass index, *IQR* interquartile range

*Cortisone medication was defined as cortisone treatment before the patient was referred for surgery

In the multivariable logistic regression analysis of risk factors for SSI in the matched case–control cohort, lymph node dissection OR 3.22 (95% CI 1.32–7.82) and the use of drains OR 1.82 (95% CI 1.04–3.18) were independent risk factors for SSI (Table 4).

**Table 4:** Multivariable analysis comparing patients with surgical site infection and control patients using data from Swedish National Registry for Endocrine Surgery and questionnaire data from participating departments

Variables	Odds ratio	Confidence interval
Thyroidectomy	1.00	
Lymph node dissection with or without thyroidectomy	3.22	1.32–7.82
Benign histology	1.00	
Malignancy	1.25	0.40–3.91
Post-operative drainage		
No	1.00	
Yes	1.82	1.04–3.18
Diabetes mellitus		
No	1.00	
Yes	0.55	0.29–1.04
Operation time (min)		
<60	1.0	
61–120	2.22	0.23–21.24
>120	3.76	0.40–34.82
Missing value	4.63	0.50–42.78
Post-operative bleeding		
No	1.00	
Yes	3.34	0.59–18.83

### Diagnosis and treatment of surgical site infection

The attending surgeon reported that 30 (27%) patients with SSI were diagnosed before discharge from the hospital whereas 53 (49%) patients were diagnosed after the date of discharge. Data were missing for 26 (24%) patients regarding when SSI was diagnosed. Among patients with SSI, 78 (72%) patients were treated with antibiotics, 3 (2%) patients did not receive antibiotics and data were missing for 28 (26%) patients. Readmission in the hospital due to SSI was reported in 36 (33%) patients.

Data from the case–control cohort showed that among the 34 patients with independent risk factors for SSI (i.e. lymph node dissection and post-operative drain) 26 (76%) patients had SSI. Since the use of drain was missing for the rest of cohort, a comparison between patients with SSI and the rest of cohort was not possible. Only 2 (8%) out of the 26 patients were treated with antibiotics prophylactically, whereas 16 (62%) patients did not receive prophylactic antibiotics. Data regarding antibiotic prophylaxis were missing in 8 (31%) patients (*p* = 0.12).

## Discussion

This study of a national cohort of 9494 patients who underwent surgery for thyroid disease reports a low risk of SSI, 1.2%.

Concomitant lymph node dissection and the use of drains were independent risk factors for SSI after thyroid surgery.

A previous study on complications after thyroid surgery using data from SQRTPA showed that patients who underwent lymph node dissection and were reoperated due to bleeding had increased risk of SSI [4]. The present study included data from three times more patients, and additional information from the attending surgeons, which enabled a nested case–control analysis to be performed. In the present study, lymph node dissection was verified as an independent risk factor for SSI. Additionally, the use of prophylactic drainage was proved to be an independent risk factor. However, reoperation for post-operative bleeding was not a risk factor.

A recent meta-analysis has shown that the use of drain in thyroid surgery is associated with high rate of SSI, prolonged hospital stay and a high pain score [18–20]. Drains neither prevented post-operative bleeding nor did they facilitate early diagnosis of bleeding [10, 19, 20]. In agreement, the present study showed that the use of drain in thyroid surgery increased the risk of SSI. The use of drains in other areas with low risk of post-operative infection, such as breast surgery, has also been associated with a higher risk of SSI [21]. Therefore, the use of drain in routine thyroid surgery should be discouraged.

Surgical site infection after modified radical neck dissection due to cancer in the neck is a major complication estimated to occur in 13–20% of patients undergoing this procedure [22, 23]. The reason for this is not clear, but could be due to prolonged operation time or the lymph node dissection per se. It might be considered that lymph node dissection may cause disruption to the immune system and reduce the local barrier for infection.

In a previous investigation, Bures et al. [1] found that the duration of operation was an independent risk factor for SSI in thyroid surgery. The duration of operation was, however, not significant in the multivariable analysis in the present investigation. It is therefore likely that the duration of operation is a dependent risk factor due to the time required for lymph node dissection.

Lymph node dissection and the use of drain were associated with higher incidence of SSI, and it may be argued that prophylactic antibiotics may reduce the risk of SSI in these patients. Results from a national multicentre retrospective observational study by De Palma et al. [17] and a randomized controlled trial by Uruno et al. [13] showed that antibiotic prophylaxis did not protect the patient from SSI in thyroid surgery. However, in the study done by De Palma it was not reported if the patients were subjected to concomitant lymph node dissection or not. In the single-centre RCT, all patients with reported SSI had post-operative drains and underwent lymph node dissection, with one exception.

The results from the questionnaire data showed that 26 patients with SSI were subjected to drains and lymph node dissection, 16 (62%) patients did not receive prophylactic antibiotics. Only 2 (8%) of patients with the two independent risk factors for SSI and diagnosed with SSI had prophylactic antibiotic treatment. Information about prophylactic antibiotics was missing for 8 (31%) patients. This difference, however, was not significant, possibly due to too few observations.

The strength of the present study is the large number of patients and that data are based on a national data set, SQRTPA, which at present covers over 90% of the thyroid operations in Sweden [24].

Some limitations of the current study are acknowledged. This nested case–control study was retrospective, per se, although identification of patients with SSI was based on registration of data in the SQRTPA. There were missing data for some variables, e.g. antibiotic prophylaxis, which may hamper analysis and the interpretation of the results. Further, in the analysis, it was not possible to differentiate between central and lateral lymph node dissections, since these data were available only for the latter part of the study period.

Accordingly, due to the low incidence of SSI in thyroid surgery it might be helpful to evaluate the effect of antibiotic prophylaxis in subgroups with independent risk factors rather than evaluating this effect in the whole cohort. It could be considered that subgroup of patients with independent risk factors might benefit from prophylactic antibiotics in thyroid surgery.

## Conclusion

The use of drains and concomitant lymph node dissection are associated independently with SSI in surgery for thyroid disease. Patients with these two risk factors constitute a subgroup in which prophylactic antibiotics might be considered. The use of drains in routine non-malignant thyroid surgery should be discouraged.
